# Characteristics of Peripheral Immune Function in Reproductive Females with Uterine Leiomyoma

**DOI:** 10.1155/2019/5935640

**Published:** 2019-10-24

**Authors:** Zhi-Qin Liu, Mei-Yin Lu, Ru-Liang Sun, Zhi-nan Yin, Bin Liu, Yang-zhe Wu

**Affiliations:** ^1^Department of Obstetrics and Gynecology, Baoan Maternal and Child Health Hospital, Jinan University, Shenzhen 518102, Guangdong, China; ^2^Department of Biobank, Baoan Maternal and Child Health Hospital, Jinan University, Shenzhen 518102, Guangdong, China; ^3^Department of Pathology, Baoan Maternal and Child Health Hospital, Jinan University, Shenzhen 518102, Guangdong, China; ^4^Zhuhai Precision Medical Center, Zhuhai People's Hospital (Zhuhai Hospital Affiliated with Jinan University), Jinan University, Zhuhai 519000, Guangdong, China; ^5^The Biomedical Translational Research Institute, Faculty of Medical Science, Jinan University, Guangzhou 510632, Guangdong, China

## Abstract

Inflammation and immunity are thought as risk factors for uterine leiomyoma; however, detailed reports on this topic are scarce. The present study aimed to analyze the characteristics of immune function and clinical significance of circulating CD4/CD8 T, NK, and *γδ* T cells in reproductive females with uterine leiomyoma. We analyzed the above-mentioned cells in 30 reproductive females with uterine leiomyoma and 68 healthy females using flow cytometry. After that, the correlation between function of immune cells and clinical phenotypes was analyzed. Compared with healthy controls, central memory (CM) CD4/CD8 T cells as well as Treg and Tfh cells were notably increased in leiomyoma patients; however, NK and *γδ* T cells were decreased in patients. Moreover, such alterations of these cells in patients with leiomyoma were associated with shorter menstrual cycles, longer menstrual period, anemia, pelvic lesions, more and larger myomas, and higher levels of CA125. Additionally, the increased Tfh1/Tfh2 ratio and Tfh17 were significantly associated with longer menstrual period, more myomas, and higher CA125 levels independent of age in patients with uterine leiomyoma. In conclusion, hallmarks of peripheral immune function are remarkably correlated with clinical phenotypes in reproductive females with uterine leiomyoma. This preliminary work may provide proof-of-concept for evaluating efficacy of treatment and prognosis of reproductive females with uterine leiomyoma with the help of quantitative analysis of peripheral immune function, which may inspire performing further investigations on the relevance of immune function with different diseases.

## 1. Introduction

Uterine leiomyoma, a type of benign neoplasm, commonly occurs among 50–60% of fertile women [1], the rate may reach 70% for women who are elder than 50 years old [2]. Among leiomyoma patients, 15–30% of cases may develop into severe symptoms, such as infertility, menorrhagia, and constipation [3]. In addition to hysterectomy, the existing therapeutic strategies mainly involve myomectomy by hysteroscopy, laparotomy or laparoscopy, uterine artery embolization, and interventions performed under radiological or ultrasound guidance, depending on patients' age and the number, size, and location of the fibroids [3]. Clinically, fibroids account for 30–50% of all hysterectomies and are associated with substantial morbidity and health care costs for women of reproductive age [1]. Therefore, uterine leiomyoma is harmful for women particularly for those of reproductive age.

Despite the high incidence of this disease, its etiology still remains largely unclear. Previous studies reported that a number of high-risk factors, including early menarche [4], lifestyle (diet, caffeine, and alcohol consumption) [5], obesity and metabolic disturbance [6], hereditary factor, and late age of first pregnancy [7], may increase the susceptibility of this tumor. Regarding pathogenesis of uterine leiomyoma, one hypothesis is that chronic inflammation favors onset, development, and recurrence of uterine fibroid [8]. Previous studies reported that abnormal expression of immune genes and decreased number and dysfunction of uterine NK cells were associated with leiomyoma risk [9, 10], as well as the aberrant blood vessel development and subfertility in patients with leiomyoma [11]. In addition to the above-mentioned factors, obesity, pregnancy, and menses also affect secretion of cytokines [12]; the latter can influence proliferation of neoplastic cells, fibrosis, and angiogenesis in uterine, which in turn sustain the formation and growth of fibroid [8]. Moreover, inflammation is an important cause of leiomyoma recurrence [8], frequently occurring in fibroid [13]: chronic inflammation is a key factor in awakening dormant tumor cells which are residual at the primary site, leading to recurrence [4, 8]. Therefore, host immunity may play a substantial role in tumorigenesis and development of uterine leiomyoma.

To date, a limited number of studies have concentrated on the relationship between peripheral immune function and fibroid. In the present study, we proposed a quantitative assessment method for host peripheral immunity using flow cytometry. For this purpose, we attempted to investigate alterations of peripheral immune function, including CD4+, CD8+ T cells, natural killer (NK) cells, and gamma delta T (*γδ* T) cells, among Chinese females with the age of 20∼40 years. The current study may provide a reference to detect underlying pathogenesis of uterine leiomyoma from the immune function aspect, which will inspire more investigation on the role of peripheral immune function in the occurrence, development, and prognosis of uterine leiomyoma.

## 2. Materials and Methods

### 2.1. Participants

In the present study, 30 patients with uterine leiomyoma who aged 20∼40 years old were enrolled at Baoan Maternal and Child Health Hospital, Jinan University (Shenzhen, China) between June 2018 and December 2018. All patients received hysteroscopy, and their diagnoses were verified by pathological examinations. At the day before operation, a 2 mL of anticlotting blood sample was collected for immune test from each case after signing the written informed consent form. Moreover, the patients' data of menstrual cycle, clinical features of myomas, the values of blood tests, and complications of leiomyoma were collected from medical records.

During the same time, 68 healthy females who aged 20∼40 years old were randomly recruited from the subjects who arrived our hospital for premarital or prepregnancy medical examinations, with exclusion of subjects with endometrial polyps, ovary morbidities, infertility, leiomyoma, and other tumors. After signing the consent form, information of the healthy controls such as age, menstrual cycle and period, and reproductive history were collected by trained nurses through a questionnaire survey, and 2 mL of venous blood was collected for flow cytometry analyses.

All the research subjects were unrelated ethnic Han Chinese population of southern China. The respond rates of both patients and controls were more than 90%.

The present study was approved by the Ethics Committee of the Baoan Maternal and Child Health Hospital, Jinan University (IRB no. LLSC-2018-02-01).

### 2.2. Flow Cytometry Analyses

Peripheral white blood cells were isolated through hemolysis by adding FACS lysing solution (BD Biosciences, San Jose, CA, USA). After the precipitates were washed twice by phosphate-buffered saline (PBS), cells were labelled according to our routine method [14]. Antibodies for PerCP-Cy5.5-conjugated anti-CD3; APC-conjugated anti-CD4; FITC-conjugated anti-CD45RA; PE-conjugated anti-CD25 and anti-V*δ*2; PE-Cy7-conjugated anti-CD28 and anti-NKG2D; BV421-conjugated anti-56, anti-127, anti-CD194, and anti-TCR*γδ*; BV510-conjugated anti-CD8 and anti-NKP46; Alexa Fluor 647-conjugated anti-CCR7 and anti-CXCR5; Alexa Fluor 484-conjugated anti-CD183, and BB515-conjugated anti-PD-1 were purchased from BD Biosciences.

Samples were run on a BD LSR Fortessa™ cell analyzer (BD Biosciences) at Shuangzhi Purui Medical Laboratory Co., Ltd. (China), and the data were analyzed using FlowJo 10.1 software (Tree Star Inc., Ashland, USA).

### 2.3. Statistical Analysis

The SPSS 25.0 software (IBM, Armonk, NY, USA) was used to analyze the data. Data were presented as *n*, proportion, median, and min/max. Mann–Whitney *U* tests were performed to analyze the differences of immune indexes between the patients and controls. The relationship of above indexes and clinical phenotypes were performed using Spearman correlation analyses. Lastly, partial correlation analysis and stratification analysis were performed to control the confounding factor age. All tests were two-sided, and the level of significance was set at 0.05. Statistical figures were produced by GraphPad Prism7.0 (GraphPad Software, USA).

## 3. Results

### 3.1. Clinical Characteristics of Patients and Healthy Controls

The clinical characteristics of patients and healthy controls were presented in Table 1. It was revealed that the patients with fibroid were older than the healthy controls (median: 36-year vs. 30-year, *P* < 0.001). Therefore, age was herein used in the latter partial correlation analysis and stratification analysis.

### 3.2. CD4+ T-Cell Differentiation Associates with Shorter Menstrual Cycle in Leiomyoma Patients

The difference in circulating T-cell population between leiomyoma patients and healthy controls was analyzed, and the results were shown in [Supplementary-material supplementary-material-1]. Compared with healthy controls, the double-negative T cells (CD4−CD8−) were significantly lowered in patients (*P*=0.034). However, the CD4+, CD8+, and double-positive T cells (CD4+CD8+) were not different between patients and healthy groups (all *P* values >0.05).

**Figure 1:**
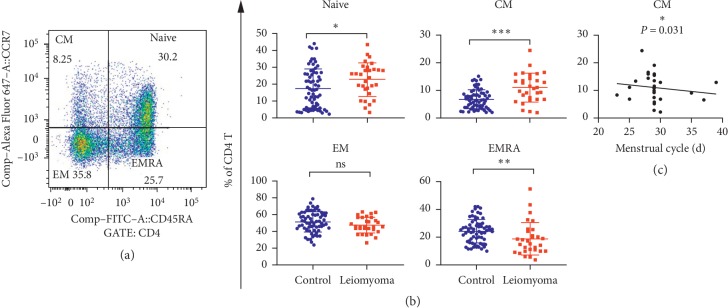
Distribution characteristics and significances of the subgroups of CD4+ cells in 30 leiomyoma patients and 68 healthy controls. (a) Flow analysis of CD4+ naïve, central memory (CM), effector memory (EM), and terminal differentiated effector memory (EMRA) cells, respectively. (b) Comparison of above cells between patients and controls. (c) Association of CD4+CM cells with menstrual cycle. ^*∗*^*P* < 0.05. ^*∗∗*^*P* < 0.01. ^*∗∗∗*^*P* < 0.001. ns, not significant.

Then, we used markers CCR7 and CD45RA to determine whether CD4+ T-cell differentiation was different between patients and healthy controls. Results are exhibited in Figure 1, showing increases of both naïve (CD4+CCR7+CD45RA+) and central memory (CM; CD4+CCR7+CD45RA−) T cells in patients with leiomyoma; meanwhile, both effector memory (EM; CD4+CCR7−CD45RA−) and CCR7−CD45RA+ terminally differentiated effector memory (TEMRA) cells were reduced in patients as compared with healthy controls (all *P* values <0.05).

We further analyzed correlations between clinical phenotypes listed in Table 1 in patients and double-negative T cells, naïve CD4+ T cells, CM CD4+ T cells, or EMRA CD4+ T cells. We found that CM CD4+ T cells rather than other CD4 subsets were reversely correlated with menstrual cycle (*r* = −0.395, *P*=0.031, Figure 1(c) and [Supplementary-material supplementary-material-1]).

### 3.3. Hallmarks of Treg, Th, and Tfh Cells in Leiomyoma Patients

**Figure 2:**
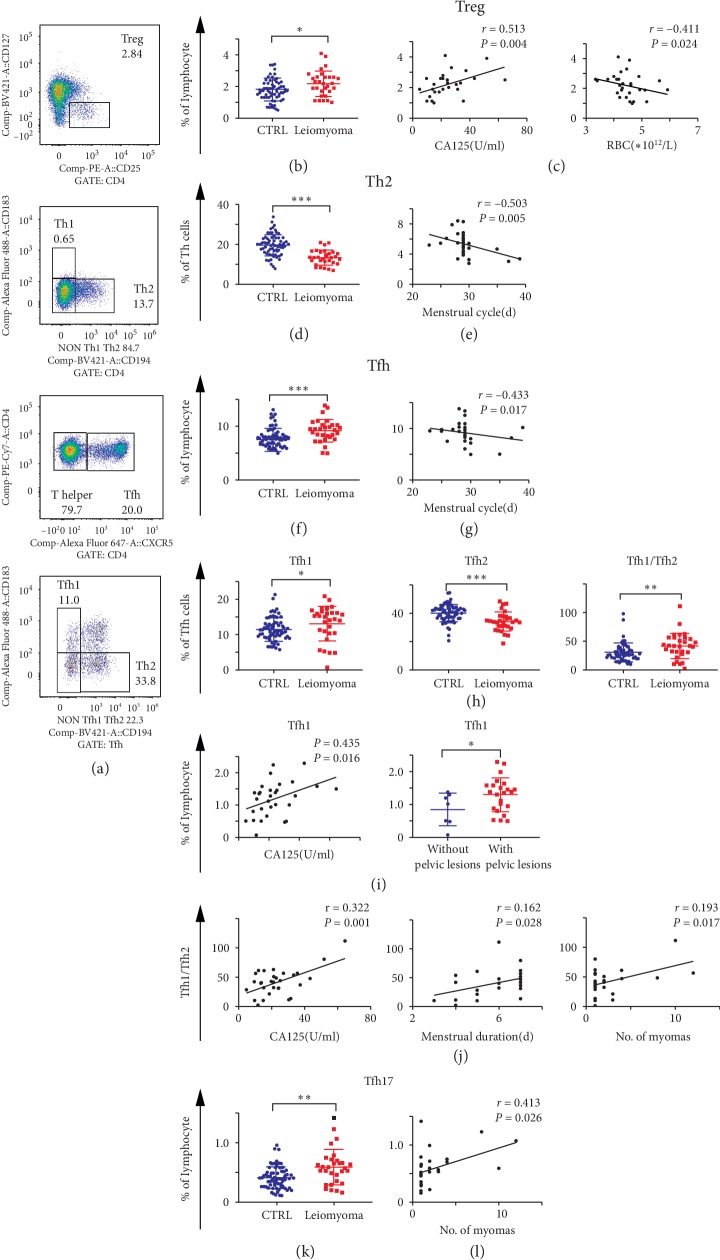
Distribution characteristics and significances of the Treg, Th, and Tfh cells in 30 leiomyoma patients and 68 controls. (a) The flow analyses of regulatory T (Treg), helper T (Th), and follicular helper T (Tfh) cells. (b, d, f, l) The comparison of above cells between patients and controls. (c) The correlations of Treg cells with CA125 and red blood cells (RBC), respectively. (e, g) The associations of Th2 and Tfh cells with menstrual cycle, respectively. (j) The association between Tfh1 cells with CA125 and pelvic diseases, respectively. (k) The association between the ratio Tfh1/Tfh2 with CA125, menstrual duration, and the numbers of myomas in each case. (l) The association between Tfh17 cells with myoma number. ^*∗*^*P* < 0.05. ^*∗∗*^*P* < 0.01. ^*∗∗∗*^*P* < 0.001. ns, not significant.

Given regulatory T (Treg; CD4+CD25+CD127−), helper T (Th; CD3+CD4+CXCR5−), and follicular helper T (Tfh; CD3+CD4+CXCR5+) cells are all differentiated from the naïve CD4+ T cells [15], we further compared the distribution of these cells between patients with uterine leiomyoma and healthy controls. As exhibited in Figure 2, compared with healthy controls, percentage of Treg, Tfh, Tfh1 (CD4+CXCR5+CXCR3+CCR4−), Tfh17 (CD4+CXCR5+CXCR3−CCR4−CCR6+) cells, and the Tfh1/Tfh2 ratio were significantly higher in patients with leiomyoma (all *P* values <0.05), while Th2 (CD4+CXCR5−CXCR3−CCR4+) and Tfh2 (CD4+CXCR5+CXCR3−CCR4+) cells decreased (both *P* values <0.001).

The associations between the above-mentioned cells and the clinical phenotypes in fibroid patients were further analyzed. As illustrated in Figure 2 and Supplementary [Supplementary-material supplementary-material-1], we found significant associations as following:

1. The amounts of Treg cells in patients positively correlated with the levels of tumor marker CA125 and negatively correlated with red blood cells (RBC) (Figure 2(c))
2. Both Th2 and Tfh cells were negatively associated with menstrual cycle (Figures 2(e)/2(g))
3. As shown in Figure 2(j), the higher levels of Tfh1 cells were observed in patients with higher CA125 levels or pelvic comorbidities
4. The Tfh1/Tfh2 ratio was positively correlated with higher CA125, longer menstrual period, and more numbers of myomas (Figure 2(k))
5. The Tfh17 cells were positively associated with the number of myomas (Figure 2(l))

### 3.4. Differentiation of CD8+ T Cells in Uterine Leiomyoma

**Figure 3:**
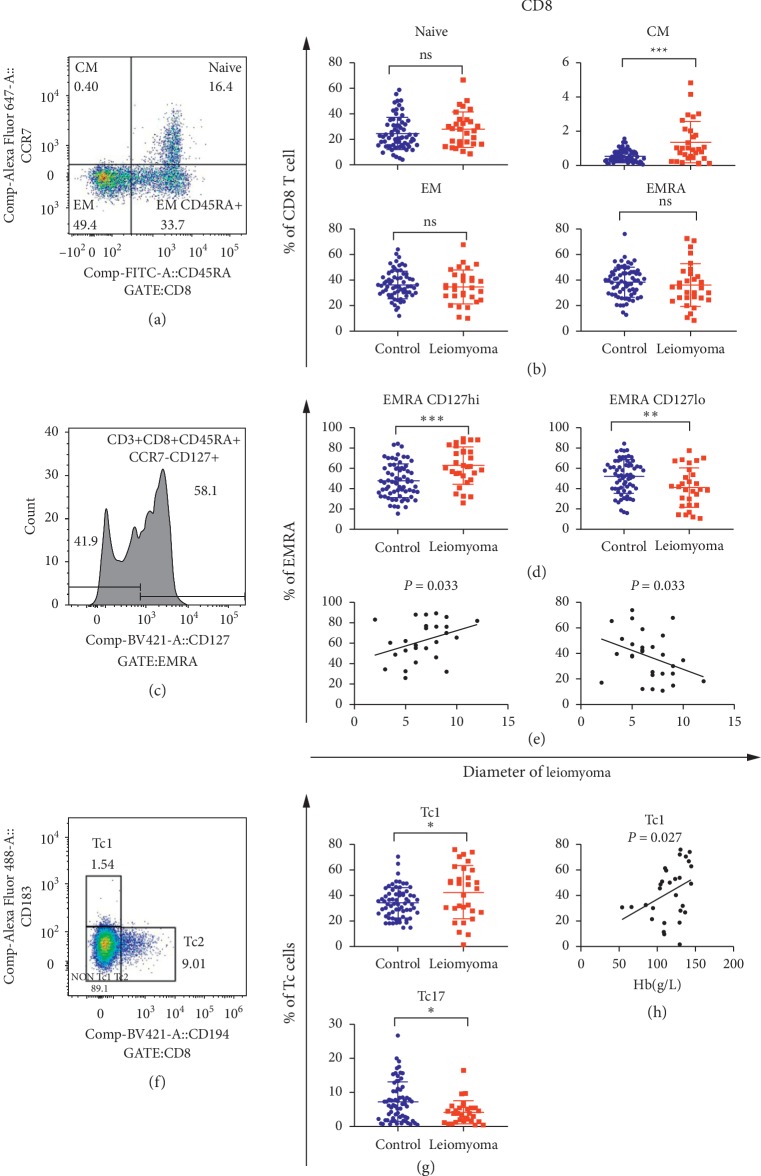
CD8+ cells, its subgroups, and diameter of leiomyoma. (a, c, f) The flow analyses of CD8+ naïve, CM, EM, EMRA, EMRA CD127^high/low^ cells, and Tc cells, respectively. (b, d, g) The comparison of above cells between 30 patients and 68 controls. (e) Association between CD8+ EMRA CD127hi/lo cells with the diameter of leiomyoma in 30 patients. (h) Association between Tc1 cells and hemoglobin (Hb) in 30 patients. ^*∗*^*P* < 0.05. ^*∗∗*^*P* < 0.01. ^*∗∗∗*^*P* < 0.001. ns, not significant.

Figure 3 illustrated that CM CD8+ cells and cytotoxic T cells (Tc1) (CD8+CXCR5−CXCR3+CXCR4−) were significantly increased in patients with uterine leiomyoma compared with healthy controls; while Tc17 cells (CD8+CXCR5−CXCR3+CXCR4−) were decreased. However, naïve, EM, and EMRA CD8 cells were not significantly different between patients and controls (all *P* values >0.05).

We further analyzed the relationship between CD8+ T-cell differentiation and clinical phenotypes. As shown in Figure 3 and [Supplementary-material supplementary-material-1], only Tc1 cells were positively correlated with higher Hb levels. In EMRA CD8+ cells, which are mainly circulated into the peripheral tissues and retained in these tissues [16], CD127^high^ expression subset cells (CD8+CCR7−CD45RA+CD127^high^) increased, while CD127^low^ expression subset cells decreased in patients (Figure 3(d)). Furthermore, increased proportion of CD127^high^ cells in EMRA CD8+ cells was associated with larger myomas in patients with leiomyoma.

### 3.5. Function Depletion of NK Cells in Leiomyoma Patients with Longer Menstrual Period and Anemia

**Figure 4:**
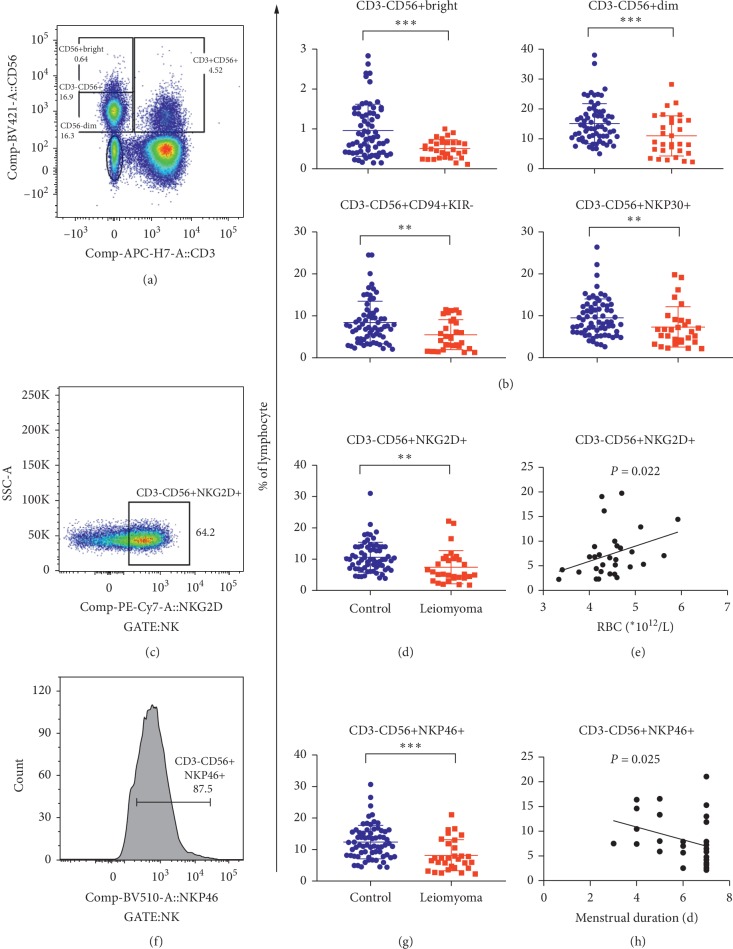
Distribution characteristics and significances of NK cells in 30 leiomyoma patients and 68 controls. (a, c, f) The flow analyses of the subgroups of the NK cells. (b, d, g) Comparison of the subgroups of the NK cells between patients and controls. (e) Correlation of CD56+NKG2D+ cells with red blood cells (RBC). (f) Correlation of CD56+NKP46+ cells with menstrual duration. ^*∗∗*^*P* < 0.01. ^*∗∗∗*^*P* < 0.001.

Since NK cells play pivotal roles in the innate immunity, functional characteristics of NK cells were also explored using flow cytometry (Figure 4 and [Supplementary-material supplementary-material-1]). As displayed in Figure 4, we found that both CD56^bright^ and CD56^dim^ cells as well as NKT cells (CD3+CD56+) were significantly reduced in peripheral blood of patients compared with healthy controls. This apparently demonstrated reduction of total amount of NK cells, implicating with impaired innate immune function. Furthermore, we revealed that functional subsets of NK cells, including CD94+KIR−, NKP30+, NKP46+, and NKG2D, significantly decreased in patients (all *P* values <0.01, Figure 4(b)). Additionally, we found positive correlation between decreases in CD56+NKG2D+ NK cells and reduction of RBC, reflecting that NK cells would be associated with anemia in leiomyoma patients (*r* = 0.417, *P*=0.022, Figure 4(d)).

### 3.6. Depressed Immune Function of *γδ* T Cells in Leiomyoma Patients

**Figure 5:**
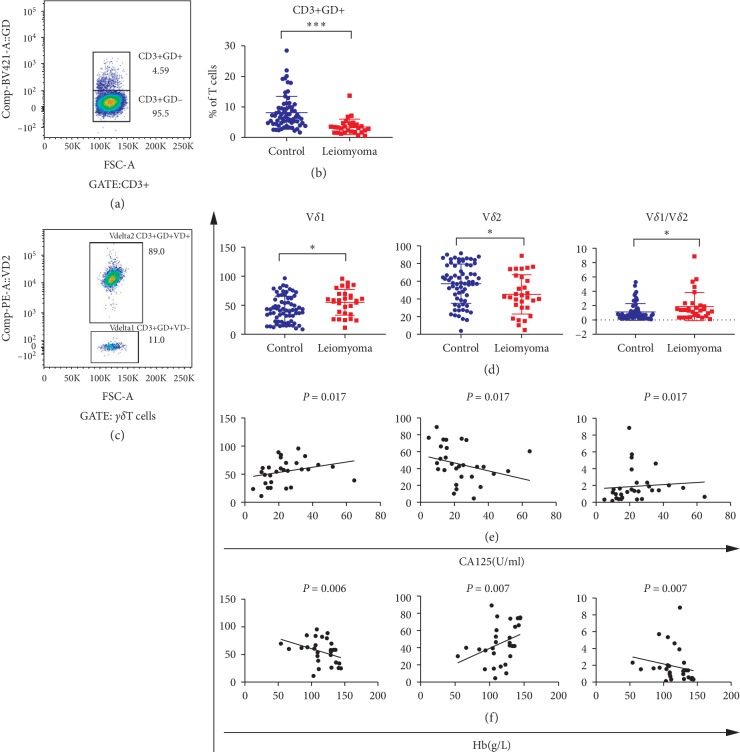
Distribution characteristics and significances of *γδ* T cells in 30 leiomyoma patients and 68 controls. (a, c) The flow analyses of *γδ* T cells, V*δ*1+, and V*δ*2+ cells, respectively. (b) The comparison of *γδ* T cells between patients and controls. (d) The comparison of V*δ*1, V*δ*2 cells, and the ratio V*δ*1/V*δ*2 between patients and controls. (e, f) The associations of V*δ*1, V*δ*2 cells, and the ratio V*δ*1/V*δ*2 with circulating CA125 and hemoglobin (Hb) level, respectively. ^*∗*^*P* < 0.05. ^*∗∗*^*P* < 0.01. ^*∗∗∗*^*P* < 0.001.

Given the unique function of *γδ* T cell in antitumor immunity, it is of significant importance to detect how *γδ* T cells were functionally altered in patients with uterine leiomyoma. Firstly, we found that, compared with healthy controls, patients have remarkably lower amount of *γδ* T cells (*P* < 0.001, Figure 5(b)). Moreover, V*δ*1 cells (immune depression subset) increased, while V*δ*2 cells (antitumor subset) decreased in patients, which led to elevation of the ratio of V*δ*1/V*δ*2 in those patients (all *P* values <0.05, Figure 5(d)). Furthermore, the findings indicated that V*δ*2+NKG2D+ *γδ* T cells were decreased, whereas V*δ*2+NKP30+ *γδ* T cells were increased in leiomyoma patients (both *P* values <0.05, [Supplementary-material supplementary-material-1] and [Supplementary-material supplementary-material-1]), which contributed to the decrease of CA125 level in patients. This was further supported by correlation analyses, exhibiting a negative correlation between V*δ*2+NKG2D+ *γδ* cells and CA125 level in leiomyoma patients.

### 3.7. Age Variation Slightly Influences Immune Function

Considering age of patients and healthy controls distributed between 26–40 and 22–36 years old, respectively, we therefore analyzed the association between age variation and above important immune function indexes (e.g., CM CD4+ T, CD8 EMRA CD127^high^, Treg, Th2, Tfh1, Tfh17, CD56+NKG2D+, V*δ*2, and V*δ*2+NKG2D+ cells) to test how age variation may affect immune function ([Supplementary-material supplementary-material-1]). We found that all of these immune indexes showed no statistical correlation with age variation in healthy controls (all *P* values >0.05); as for patient group, only CM CD4+ T cells showed a correlation with age (*r* = −0.456, *P*=0.011). Therefore, these results suggested that age variation was not a main factor affecting immune function in the context of this study.

Additionally, we further evaluated the relationship between immune function indexes and clinical phenotypes using partial correlation analysis. We found that the increased Tfh1/Tfh2 ratio remained significantly correlated with longer menstrual period, more myomas, and higher CA125 levels in patients with uterine leiomyoma, and Treg, Tfh17, and V*δ*2+PD−1 cells showed a notable correlation with higher CA125 and more myomas as well (Table 2, all *P* values <0.05).

## 4. Discussion

Currently, little is known on how peripheral immune cell function alters in the context of diseases because the existing clinical blood tests provide only ratios of a certain types of immune cells, such as CD3+, CD4+, CD8+, monocytes, and neutrophils. However, such examinations do not give a whole picture or clues on how these immune cells may influence immune function. In the present study, we evaluated hallmarks of peripheral immune cell function of reproductive females with uterine leiomyoma.

Firstly, we assessed T-cell function and found that CD4−CD8− T cells were significantly decreased in fibroid patients in this study. There are three subtypes of CD4−CD8− T cells, which are infrequent in the peripheral blood, including *γδ* T cells, a subset of NKT cells, and double-negative T cells from the thymus [17]. In the current research, we did not find a significant difference in the levels of NKT cells between patients with uterine leiomyoma and healthy controls (data were not shown). Combing the findings of the *γδ* T cells, it could be concluded that the reduced levels of CD4−CD8− T cells might reflect the depressed function of *γδ* T cells in fibroid patients in this study.

Then, we analyzed CD4+ T-cell function and found that CD4+ T-cell differentiation was modulated, including an increase of naïve CD4+ T cells. The naïve CD4+ T cells can differentiate into Treg, Tfh, and Th cells [15]; therefore, these cells were further analyzed in the present study. We found that Treg cells were also unregulated in fibroid patients with higher CA125 levels, even after controlling age as a confounding factor. Treg cell is a type of immunosuppressive T-cell population, preventing autoimmunity and maintaining immunological tolerance [18]. Infiltration of a large number of Treg cells into tumor tissues is often associated with tumor development and poor prognosis [19], while removal of Treg cells is able to evoke and enhance antitumor immune response [20, 21]. Thus, it is plausible that Treg cells increased in leiomyoma patients with higher levels of tumor markers in this study. Combing our findings and above published reports, Treg cells can serve as a biomarker for leiomyoma development.

In addition, Tfh cells and subgroups (Tfh1 and Tfh17 cells) were also unregulated in the patients with uterine leiomyoma, associating with longer menstrual periods, higher CA125 levels, more myomas, or with pelvic lesions in this study. With controlling age as a confounding factor, Tfh cells and their subgroups were herein found as independent risk factors for uterine leiomyoma. The role of Tfh cells in pathology of fibroid is still elusive. These cells are critical for activation of B cells, antibody class switching, and germinal center (GC) formation [22]. Recent analyses of human blood cells identified major functional subsets (Tfh1, Tfh2, and Tfh17), with specific Tfh subsets correlated with broad neutralizing antiviral Ab or auto-Ab [23]. Activation of Tfh cell response is essential for infection clearance, whereas unregulated Tfh-mediated immune response is implicated in inflammation progression [9, 10, 24], as a risk factor for uterine leiomyoma [8]. Gu-Trantien et al. found that Tfh cells may be a key factor in immune suppression of antitumor responses in the chronic inflammatory microenvironment [25], suggesting that Tfh cells are correlated with tumor progression. Combing these reports and our data, the findings of the association of the Tfh1/Tfh2 ratio with more myomas and higher values of CA125 are credible, so as the relation between Tf17 and more myomas. Moreover, the function of Tfh cells was reported to be mediated by exposure to sex hormone in mice [26] and fish [27]. The relationship of Tfh cells with longer menstrual period could be explained via the immune dysfunction by hormonal exposure. Thus, Tfh cells and their subgroups are feasible indexes for progression of uterine leiomyoma and severity of symptoms.

Moreover, a decrease in Th2 cells was found in the patients with longer menstrual periods in the current study. Th2 cells secret a large amount of cytokines, such as IL4 in the luteal phase [28], which are characteristics of extracellular defense as well as self-cell tolerance [29]. Th2 bias is observed in premenarche, postmenopause, and pregnancy, which wholly contribute to attenuate risk of uterine leiomyoma [30]. Therefore, reduced Th2 levels in fibroid patients are plausible in this study. In addition, estrogen replacement therapy [31] and high progesterone/estradiol ratio [32] in luteal phase or pregnancy may affect the function of Th2 cells. Thus, it is plausible that Th2 cells are associated with aberrant menstrual cycles in this study. Our findings highlighted Th2 cell as a biomarker for hormone therapy in uterine leiomyoma.

Furthermore, the present study indicated that CD8+CCR7−CD45RA+CD127^high^ cells, a subgroup of CD8 memory cells, were associated with larger myomas. CD127 is the alpha chain of the IL-7 receptor located in CD8 cells and is involved in the survival, homeostasis, and function of T cells [33, 34], especially the memory T-cell mediating chronic inflammatory diseases [35]. Our data on CD8+CCR7−CD45RA+CD127^high^ cells were consistent with the theory that chronic inflammation is a risk factor for fibroid [8]. We also found CD4/CD8 CM cells increased in patients with uterine leiomyoma. In addition, CD4 CM cells were associated with aberrant menstrual cycles. However, this association was not independent after adjusting for age as a confounding factor. The circulating CM T cells contribute to the maintenance of peripheral memory T-cell populations for response to a secondary pathogen challenge [16, 36], thereby rapidly clearing pathogen [16]. Based on the fact that chronic infections are related to the etiology of leiomyoma [9, 10], our results on the association between CD4/CD8 CM cells and leiomyoma seem to be biologically plausible. It can be concluded that CD4/CD8 CM cells and CD8+CCR7−CD45RA+CD127^high^ cells can be served as inflammation markers for uterine leiomyoma.

Finally, we found that the decrease of cytotoxic cells, such as Tc1, CD56+NKG2D+ NK, and V*δ*2+NKG2D+ *γδ* T cells, were related to anemia in fibroid patients. NKG2D is a receptor expressed on the surface of all human NK, *γδ*, and CD8 T cells [37]. Several mouse models [38–40] and *in vitro* studies [41] showed antitumor cytotoxicity of NKG2D receptor in NK and *γδ* T cells. The expression of this receptor associates with aberrant blood vessel development in uterine [11]; therefore, it is feasible that, in the present study, the anemia of fibroid patients might associate with endometrial bleeding related to dysfunction of NKG2D [42]. In addition, Tc1 cells (IFN*γ*-producing CD8+ T cells) promote dysfunction of hematopoietic stem cell and depletion of myeloid lineage progenitor cells, resulting in anemia [43–45]. Combing these reports, our findings suggested that the above-mentioned cytotoxic immune functions are markers for severe anemia in uterine leiomyoma.

Above findings have clinical significance and relevance. Longer menstrual period and anemia correlate with abnormal bleeding in leiomyoma patients [3]. A higher CA125 level typically represents tumor progression [46]. Larger diameter of tumors has been found as a predictor for massive intraoperative hemorrhage during caesarean delivery [47]. Furthermore, these symptoms may affect the selection of a therapeutic approach [3] and are related to fibroid recurrence [48]. Therefore, our findings implied a prospect for the prevention and treatment of fibroids. For example, unregulated Treg and Tfh cells demonstrate a possibility of further development of fibroid with pelvic masses or pelvic pain, representing the necessity to surgical interventions. The decrease of cytotoxic cells, such as Tc1, CD56+NKG2D+ NK, and V*δ*2+NKG2D+ *γδ* T cells, can also serve as biomarkers for surgery, predicting future severe anemia. In addition, CD8+CCR7−CD45RA+CD127^high^ cells associate with larger myomas, increasing the risk for obstetric complications, and therefore need to be solved before pregnancy. Moreover, patients with higher Treg, Tfh, and CD8+CCR7−CD45RA+CD127^high^ cells have higher risk for fibroid recurrence because of more and larger myomas.

## 5. Conclusions

Our present study for the first time attempted to reveal hallmarks of peripheral immune function characteristics in reproductive females with uterine leiomyoma. Our results demonstrated that compared with healthy controls, CD4/CD8 CM, Treg, and Tfh cells were significantly increased and that functional NK and *γδ* T cells were notably attenuated in patients with uterine leiomyoma. Moreover, the increased ratio Tfh1/Tfh2, Treg, and Tfh17 cells contributed to longer menstrual period, more myomas, and higher levels of tumor marker CA125. The present research may provide a proof-of-concept for exploring underlying cross-talks between peripheral immune function and clinical phenotypes of reproductive females with uterine leiomyoma.

## Figures and Tables

**Table 1:** Clinical and demographic characteristics of uterine fibroid patients and fibroid-free controls.

Variables	Patients (*n* = 30)	Controls (*n* = 68)	*P* (Mann–Whitney *U*)
Age (years)	36 (26, 40)	30 (22, 36)	<0.001
Menstrual cycle (days)	29 (23, 39)	30 (22, 45)	0.003
Menstrual duration (days)	7 (3, 7)	6 (5, 8)	0.357
No. of myomas	1 (1, 12)	—	
1 (*n*, %)	18 (60.0%)	—	
2 (*n*, %)	5 (16.7%)	—	
≥3 (*n*, %)	7 (23.3%)	—	
Site of myoma^1^			
Intramural (*n*, %)	24 (80%)	—	
Subserous (*n*, %)	3 (10%)	—	
Intraligamentary (*n*, %)	2 (6.7%)	—	
Submucous (*n*, %)	1 (3.3%)	—	
Diameter of myoma^1^ (cm)	7 (2, 12)	—	
≤5.0 (*n*, %)	9 (30%)	—	
>5.0 (*n*, %)	21 (70%)	—	
Complications			
Pelvic lesions (*n*, %)	23 (76.7%)	—	
Salpingitis (*n*, %)	16 (53.3%)	—	
Blood tests			
CA125 (U/ml)	21.2 (4.8, 95.9)	—	
Hb (g/L)	119.5 (54, 144)	134 (116, 154)	<0.001
RBC (10^12^/L)	4.45 (3.33, 5.92)	4.42 (2.53, 5.26)	0.638

Data were presented as number, median, min/max, or percentage. ^1^The characteristics of the biggest myoma; Hb, hemoglobin; RBC, red blood cells.

**Table 2:** Partial correlation analysis and stratification analysis controlling the confounding factor age (*n* = 30).

Clinical phenotypes/immune indexes	Uncontrolled Spearman correlation	Controlled^3^ partial correlation	≤35 stratification analysis	>35 stratification analysis
Menstrual cycle, days				
CD4CM (% of CD4 T cell)	−0.395, 0.031	−0.005, 0.979	−0.306, 0.309	−0.240, 0.353
Th2 (% of Th cells)	−0.488, 0.006	−0.367, 0.051	−0.163, 0.595	−**0.731, 0.001**
Tfh (% of lymphocyte)	−0.433, 0.017	−0.280, 0.226	−0.550, 0.051	−0.425, 0.089

Menstrual duration, days				
Tfh1/Tfh2	0.389, 0.028	**0.375, 0.045**	0.032, 0.918	0.461, 0.062
CD56+NKP46+ (% of lymphocyte)	−0.409, 0.025	−0.295, 0.121	−0.218, 0.474	−0.375, 0.139

Number of myomas				
Tfh1/Tfh2	0.260, 0.017	**0.437, 0.020**	0.081, 0.768	0.556, 0.250
Tfh17 (% of lymphocyte)	0.413, 0.026	**0.445, 0.018**	0.504, 0.079	0.265, 0.322

Diameter of myoma^1^				
CD8EMRACD127^high^ (% of CD8 EMRA)	0.390, 0.033	0.350, 0.063	0.302, 0.316	0.471, 0.056

Pelvic lesions (Mann–whitney *U*)^2^				
Tfh1 (% of lymphocyte)	−2.061, 0.037	—	−0.268, 0.789	−1.860, 0.063

RBC				
Treg (% of lymphocyte)	−0.411, 0.024	−0.286, 0.132	−0.407, 0.167	−0.430, 0.085
CD56+NKG2D+ (% of lymphocyte)	0.417, 0.022	0.209, 0.276	0.305, 0.310	0.314, 0.220

Hb				
Tc1 (% of Tc)	0.405, 0.027	0.360, 0.055	0.556, 0.049	0.292, 0.255
V*δ*2 (% of *γδ* T cells)	0.482, 0.007	0.307, 0.106	0.531, 0.062	0.300, 0.242
V*δ*2+NKG2D+ (% of lymphocyte)	0.373, 0.042	0.090, 0.641	0.314, 0.297	0.314, 0.219
V*δ*1/V*δ*2	−0.482, 0.007	−0.135, 0.485	−0.547, 0.053	−0.303, 0.237

CA125				
Treg (% of lymphocyte)	0.513, 0.005	**0.449, 0.015**	0.351, 0.239	**0.580, 0.015**
Tfh1/Tfh2	0.340, 0.001	**0.543, 0.002**	0.281, 0.353	0.349, 0.169
V*δ*1/Vδ2	0.431, 0.017	0.022, 0.909	0.055, 0.859	**0.608, 0.010**
V*δ*2+NKG2D+ (% of lymphocyte)	−0.363, 0.049	−0.294, 0.122	−0.198, 0.517	−0.423, 0.091
V*δ*2+PD1+ (% of lymphocyte)	−0.381, 0.038	−**0.310, 0.102**	−0.071, 0.817	−**0.586, 0.013**

Data were presented as correlation coefficients (*r*) and *P* values. ^1^The characteristics of the biggest myoma. ^2^*Z* and *P* values of Mann–whitney *U* test. ^3^Adjusted for age. Hb, hemoglobin; RBC, red blood cells.

## Data Availability

The raw data on clinical phenotypes and laboratory tests used to support the findings of this study are available from the corresponding author upon request.

## Supplementary Materials

Supplementary Materials Figure S1: the different distributions of circulating T cells in leiomyoma patients and controls. Figure S2: V*δ*2 subgroups and laboratory indexes in leiomyoma patients. Table S1: the different distributions of immune indexes in fibroid patients and healthy controls and their association with clinical phenotypes in patients. Table S2: association of age with the important immune indexes in leiomyoma patients and controls.
