# Supplementary material for: Characteristics of Peripheral Immune Function in Reproductive Females with Uterine Leiomyoma
Source: J Oncol. 2019 Oct 24;2019:5935640. doi: 10.1155/2019/5935640 (PMC6854963; doi:10.1155/2019/5935640)
Supplement: Supplementary Materials — Figure S1: the different distributions of circulating T cells in leiomyoma patients and controls. Figure S2: Vδ2 subgroups and laboratory indexes in leiomyoma patients. Table S1: the different distributions of immune indexes in fibroid patients and healthy controls and their association with clinical phenotypes in patients. Table S2: association of age with the important immune indexes in leiomyoma patients and controls. [file 5935640.f1.zip › 5935640.f1/Table S1-2.docx]

**Supplementary Table 1 The different distribution of immune indexes in fibroid patients and healthy controls, and their association with clinical phenotypes in patients**

| Variables | Patients vs  Controls  (*Z,P)^a^* | Associations with clinical phenotypes in patients (*r,P*)*^b^* | | | | | | | With or without  pelvic lesions in patients  (*Z,P)^a^* | |
| --- | --- | --- | --- | --- | --- | --- | --- | --- | --- | --- |
|  |  | Menstrual  cycle | Menstrual  duration | No. of  myomas | Diameter of  myoma | RBC | Hb | CA125 |  |  |
| CD3+CD4+  (% of T cells) | -1.950,0.051 | -0.013,0.946 | -0.328,0.076 | 0.329,0.081 | 0.049,0.796 | <0.001,1.000 | 0.072,0.704 | 0.140,0.459 | -0.687,0.492 | |
| CD3+CD8+  (% of T cells) | -1.426,0.154 | -0.171,0.365 | 0.045,0.814 | -0.352,0.061 | 0.143,0.452 | 0.074,0.696 | -0.239,0.204 | -0.022,0.908 | -1.103,0.270 | |
| CD4+CD8+  (% of T cells) | -0.763,0.445 | 0.209,0.268 | -0.352,0.057 | -0.120,0.534 | 0.072,0.706 | -0.349,0.059 | -0.057,0.765 | -0.015,0.938 | -0.539,0.590 | |
| CD4-CD8-  (% of T cells) | **-2.120,0.034** | 0.068,0.722 | 0.262,0.162 | -0.273,0.151 | -0.118,0.535 | -0.058,0.762 | 0.126,0.507 | -0.108,0.569 | -0.098,0.922 | |
| CD4 Naive  (% of CD4 T cell) | **-2.313,0.021** | -0.262,0.162 | 0.104,0.584 | 0.187,0.330 | -0.093,0.624 | -0.139,0.463 | -0.108,0.569 | 0.295,0.113 | -0.049,0.961 | |
| CD4 CM  (% of CD4 T cell) | **-3.823,<0.001** | **-0.395,0.031** | 0.243,0.195 | -0.008,0.966 | -0.065,0.731 | -0.146,0.443 | -0.291,0.119 | 0.238,0.206 | -0.270,0.787 | |
| CD4 EM  (% of CD4 T cell) | -1.665,0.096 | 0.173,0.359 | 0.173,0.361 | -0.414,0.026 | -0.030,0.876 | -0.102,0.590 | -0.049,0.795 | -0.437,0.016 | -0.172,0.864 | |
| CD4 EMRA  (% of CD4 T cell) | **-3.810,0.001** | 0.309,0.097 | -0.289,0.121 | 0.127,0.512 | 0.197,0.296 | 0.200,0.289 | 0.349,0.059 | -0.107,0.573 | -0.172,0.864 | |
| Treg  (% of lymphocyte) | **-2.239,0.025** | -0.126,0.507 | -0.026,0.893 | 0.001,0.997 | -0.291,0.118 | **-0.411,0.024** | -0.173,0.360 | **0.513,0.005** | -0.345,0.737 | |
| Th1  (% of Th cells) | -0.031,0.975 | 0.219,0.245 | 0.485,0.007 | -0.005,0.978 | -0.046,0.808 | 0.020,0.916 | 0.080,0.673 | -0.210,0.266 | -1.447,0.148 | |
| Th2  (% of Th cells) | **-5.254,<0.001** | **-0.488,0.006** | -0.239,0.204 | -0.259,0.174 | 0.093,0.626 | -0.226,0.230 | -0.187,0.323 | 0.038,0.843 | -0.638,0.524 | |
| Th17  (% of Th cells) | -0.786,0.432 | -0.304,0.103 | -0.154,0.417 | -0.180,0.351 | -0.124,0.512 | -0.137,0.469 | -0.332,0.073 | 0.082,0.667 | -0.638,0.524 | |
| Tfh  (% of lymphocyte) | **-3.326,<0.001** | **-0.433,0.017** | 0.036,0.848 | 0.090,0.642 | 0.058,0.763 | -0.130,0.495 | -0.035,0.853 | 0.097,0.610 | -0.638,0.524 | |
| Tfh1  (% of lymphocyte) | **-2.702,0.007** | -0.041,0.831 | 0.391,0.033 | 0.342,0.069 | -0.177,0.349 | 0.109,0.565 | 0.001,0.994 | 0.435,0.016 | **-2.061,0.037** | |
| Tfh2  (% of lymphocyte) | -0.162,0.871 | -0.426,0.019 | -0.142,0.454 | 0.085,0.662 | 0.087,0.647 | -0.280,0.133 | 0.057,0.765 | 0.091,0.633 | -0.270,0.787 | |
| Tfh17  (% of lymphocyte) | **-3.115,0.002** | -0.310,0.095 | 0.047,0.806 | **0.413,0.026** | -0.115,0.546 | -0.196,0.299 | 0.052,0.784 | 0.240,0.201 | -0.908,0.364 | |
| Tfh1/Tfh2 | **-3.068,0.002** | 0.188,0.319 | **0.389,0.028** | **0.260,0.017** | -0.337,0.068 | 0.109,0.566 | -0.043,0.820 | **0.340,0.001** | -0.711,0.501 | |
| CD8 Naive  (% of CD8 T cell) | -1.056,0.291 | -0.365,0.047 | 0.210,0.266 | 0.154,0.426 | 0.073,0.703 | -0.247,0.188 | -0.169,0.372 | 0.347,0.060 | -0.049,0.961 | |
| CD8 CM  (% of CD8 T cell) | **-3.562,<0.001** | -0.236,0.209 | 0.058,0.763 | 0.001,0.995 | -0.163,0.389 | -0.321,0.084 | -0.106,0.578 | 0.274,0.142 | -0.588,0.556 | |
| CD8 EM  (% of CD8 T cell) | -0.682,0.495 | -0.027,0.886 | 0.220,0.242 | -0.207,0.280 | -0.258,0.169 | -0.197,0.296 | 0.114,0.549 | -0.105,0.581 | -0.074,0.941 | |
| CD8 EMRA  (% of CD8 T cell) | -1.218,0.223 | 0.360,0.051 | -0.376,0.041 | 0.083,0.668 | 0.237,0.207 | 0.419,0.021 | 0.104,0.584 | -0.275,0.141 | -0.589,0.556 | |
| CD8EMRA CD127hi  (% of CD8 EMRA) | **-3.523,<0.001** | 0.116,0.542 | -0.247,0.187 | -0.121,0.533 | **0.390,0.033** | 0.258,0.169 | -0.280,0.134 | -0.139,0.465 | -0.368,0.713 | |
| Tc1  (% of Tc cells) | **-2.024,0.043** | 0.161,0.396 | 0.331,0.074 | 0.249,0.194 | 0.028,0.883 | 0.291,0.119 | 0.405,0.027 | -0.011,0.954 | -1.397,0.162 | |
| Tc2  (% of Tc cells) | -1.869,0.062 | 0.030,0.874 | -0.342,0.065 | -0.312,0.099 | -0.146,0.440 | -0.446,0.014 | -0.302,0.104 | -0.143,0.450 | -1.054,0.292 | |
| Tc17  (% of Tc cells) | **-2.525,0.012** | -0.084,0.660 | 0.057,0.763 | -0.265,0.165 | -0.203,0.283 | -0.226,0.229 | -0.324,0.081 | 0.006,0.977 | -0.809,0.418 | |
| CD56+bright  (%of lymphocyte) | **-3.315,<0.001** | 0.007,0.972 | 0.078,0.683 | 0.087,0.653 | -0.025,0.897 | -0.143,0.452 | -0.232,0.217 | 0.031,0.870 | -1.546,0.122 | |
| CD56+dim  (%of lymphocyte) | **-2.767,0.006** | 0.023,0.904 | -0.240,0.202 | -0.069,0.721 | 0.223,0.236 | 0.321,0.084 | -0.015,0.938 | -0.227,0.227 | -0.319,0.750 | |
| CD56+KIR-  (%of lymphocyte) | **-2.752,0.006** | -0.300,0.107 | -0.317,0.088 | 0.036,0.852 | 0.184,0.330 | 0.122,0.522 | 0.092,0.627 | -0.105,0.581 | -0.466,0.641 | |
| CD56+NKP30+  (%of lymphocyte) | **-2.652,0.008** | 0.147,0.438 | -0.273,0.145 | -0.136,0.481 | 0.239,0.204 | 0.275,0.142 | -0.020,0.918 | -0.193,0.308 | -0.662,0.508 | |
| CD56+NKP46+  (%of lymphocyte) | **-3.773,<0.001** | 0.210,0.266 | **-0.409,0.025** | -0.070,0.717 | 0.297,0.110 | 0.343,0.063 | 0.066,0.731 | -0.274,0.143 | -0.025,0.980 | |
| CD56+NKG2D+  (%of lymphocyte) | **-3.392,<0.001** | -0.047,0.804 | -0.251,0.182 | -0.126,0.514 | 0.285,0.126 | **0.417,0.022** | -0.007,0.972 | -0.283,0.129 | -0.270,0.787 | |
| γδT  (% of T cells) | **-5.288,<0.001** | 0.152,0.424 | 0.016,0.933 | -0.177,0.358 | 0.020,0.916 | -0.039,0.839 | 0.138,0.467 | -0.293,0.117 | -0.760,0.447 | |
| Vδ1  (% of γδT cells) | **-2.424,0.015** | -0.160,0.400 | 0.050,0.791 | 0.129,0.504 | 0.145,0.446 | -0.210,0.265 | **-0.488,0.006** | **0.431,0.017** | -0.981,0.335 | |
| Vδ2  (% of γδT cells) | **-2.436,0.015** | 0.154,0.418 | -0.046,0.810 | -0.133,0.491 | -0.146,0.443 | 0.206,0.274 | **0.482,0.007** | **-0.433,0.017** | -0.956,0.339 | |
| Vδ1/ Vδ2 | **-2.428,0.015** | -0.157,0.409 | 0.050,0.791 | 0.129,0.504 | 0.146,0.443 | -0.211,0.264 | **-0.482,0.007** | **0.431,0.017** | -0.932,0.360 | |
| Vδ1+NKG2D+  (%of lymphocyte) | **-4.371,<0.001** | -0.023,0.903 | 0.068,0.723 | -0.163,0.402 | 0.117,0.539 | -0.188,0.320 | -0.216,0.251 | -0.024,0.898 | -0.172,0.864 | |
| Vδ1+PD1+  (%of lymphocyte) | **-3.569,<0.001** | -0.169,0.372 | -0.279,0.136 | -0.086,0.658 | -0.069,0.718 | -0.122,0.520 | -0.276,0.139 | 0.041,0.831 | -0.490,0.624 | |
| Vδ1+NKP30+  (%of lymphocyte) | -1.353,0.176 | -0.157,0.407 | 0.222,0.238 | -0.267,0.162 | -0.084,0.660 | 0.123,0.518 | -0.093,0.626 | 0.069,0.718 | -0.270,0.787 | |
| Vδ1+NKP46+  (%of lymphocyte) | -1.276,0.202 | 0.238,0.205 | -0.206,0.276 | -0.121,0.532 | 0.410,0.024 | 0.137,0.471 | -0.070,0.715 | -0.220,0.243 | -0.123,0.902 | |
| Vδ2+NKG2D+  (%of lymphocyte) | **-4.371,<0.001** | 0.168,0.376 | 0.041,0.830 | -0.220,0.251 | -0.068,0.722 | 0.082,0.667 | **0.373,0.042** | **-0.363,0.049** | -0.760,0.447 | |
| Vδ2+PD1+  (%of lymphocyte) | **-3.688,<0.001** | 0.238,0.205 | -0.053,0.783 | -0.323,0.087 | -0.011,0.955 | 0.234,0.214 | 0.332,0.073 | **-0.381,0.038** | -0.074,0.941 | |
| Vδ2+NKP30+  (%of lymphocyte) | -0.924,0.355 | 0.170,0.369 | 0.052,0.786 | -0.251,0.189 | 0.191,0.311 | 0.381,0.038 | 0.250,0.182 | -0.211,0.263 | -0.594,0.552 | |
| Vδ2+NKP46+  (%of lymphocyte) | -0.938,0.348 | 0.255,0.175 | -0.068,0.722 | -0.194,0.313 | 0.245,0.191 | 0.274,0.142 | 0.337,0.068 | -0.397,0.030 | -0.025,0.980 | |
| Notes: ^a^ Manny-Whitney U test; ^b^ Spearman Correlation analysis. | | | | | | | | | |  |

| **Supplementary Table 2 Association of age with the important immune indexes in leimyoma patients and controls**. | | |
| --- | --- | --- |
| Variables | Leiomyoma | Control |
|  | *r, P^a^* | *r, P^a^* |
| CD4CM(% of CD4 T cell) | **-0.456,0.011** | 0.166,0.175 |
| Treg(% of lymphocyte) | -0.153,0.418 | -0.065,0.599 |
| Th2(% of Th cells) | -0.282,0.131 | 0.095,0.442 |
| Tfh1(% of lymphocyte) | -0.190,0.315 | -0.028,0.822 |
| Tfh17(% of lymphocyte) | 0.107,0.573 | 0.026,0.836 |
| CD8 EMRA CD127hi  (% of CD8 EMRA) | 0.186,0.325 | -0.043,0.727 |
| Tc1 (% of Tc) | 0.116,0.543 | 0.130,0.291 |
| CD56+NKG2D+  (%of lymphocyte) | -0.036,0.852 | -0.177,0.150 |
| CD56+NKP46+  (%of lymphocyte) | 0.176,0.352 | -0.182,0.137 |
| Vδ2 (% of γδT cells) | 0.283,0.130 | 0.108,0.383 |
| Vδ2+NKG2D+  (% of lymphocyte) | 0.138,0.468 | 0.182,0.138 |
| ^a^ Spearman correlation analyses. | | |
