# Supplementary figures and images for: Characteristics of Peripheral Immune Function in Reproductive Females with Uterine Leiomyoma
Source: J Oncol. 2019 Oct 24;2019:5935640. doi: 10.1155/2019/5935640 (PMC6854963; doi:10.1155/2019/5935640)

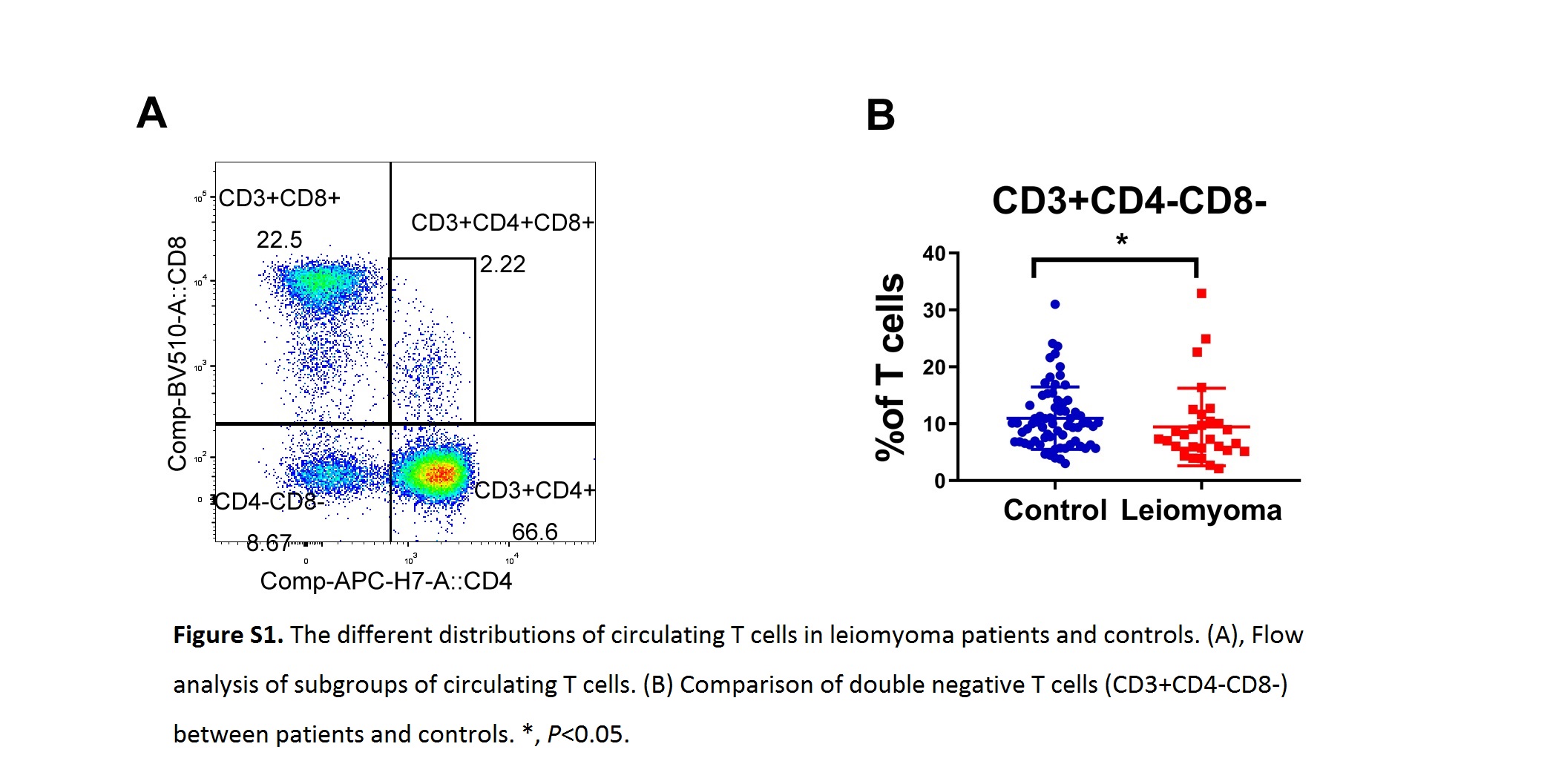

Supplement: Supplementary Materials — Figure S1: the different distributions of circulating T cells in leiomyoma patients and controls. Figure S2: Vδ2 subgroups and laboratory indexes in leiomyoma patients. Table S1: the different distributions of immune indexes in fibroid patients and healthy controls and their association with clinical phenotypes in patients. Table S2: association of age with the important immune indexes in leiomyoma patients and controls. [file 5935640.f1.zip › 5935640.f1/Figure 1s-new.jpg]

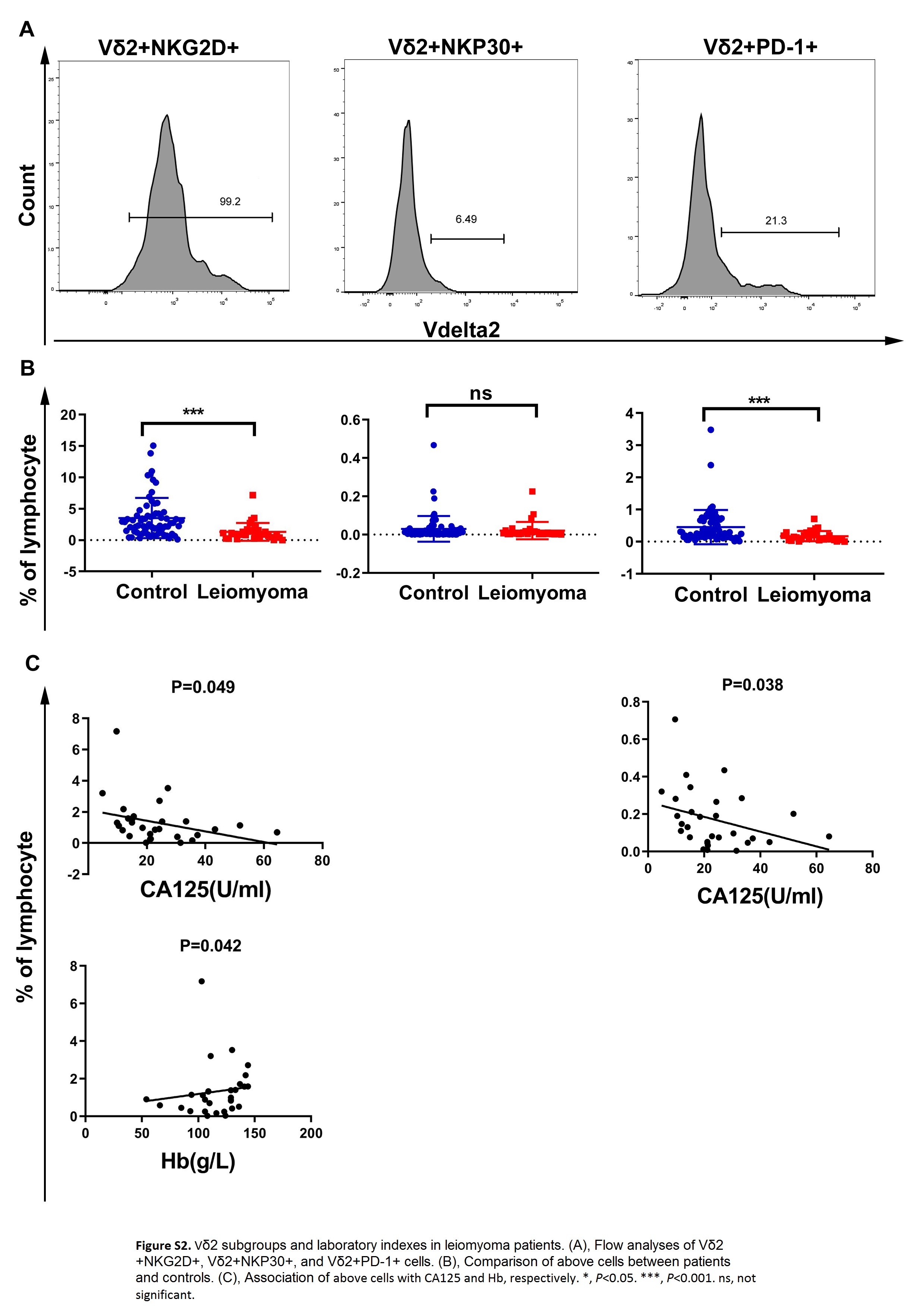

Supplement: Supplementary Materials — Figure S1: the different distributions of circulating T cells in leiomyoma patients and controls. Figure S2: Vδ2 subgroups and laboratory indexes in leiomyoma patients. Table S1: the different distributions of immune indexes in fibroid patients and healthy controls and their association with clinical phenotypes in patients. Table S2: association of age with the important immune indexes in leiomyoma patients and controls. [file 5935640.f1.zip › 5935640.f1/Figure 2s-new.jpg]
